# Comparative transcriptomic analysis reveals the roles of ROS scavenging genes in response to cadmium in two pak choi cultivars

**DOI:** 10.1038/s41598-017-09838-2

**Published:** 2017-08-23

**Authors:** Rugang Yu, Yunshu Tang, Caifeng Liu, Xueling Du, Chunmei Miao, Gangrong Shi

**Affiliations:** College of Life Sciences, Huaibei Normal University, Huaibei, Anhui 235000 P.R. China

## Abstract

To identify key regulatory genes involved in ROS scavenging in response to cadmium (Cd) exposure in pak choi, eight cDNA libraries from Cd-treated and Cd-free roots of two cultivars, Baiyewuyueman (high Cd accumulator) and Kuishan’aijiaoheiye (low Cd accumulator), were firstly performed by RNA-sequencing. Totally 0.443 billion clean reads and 244,190 unigenes were obtained from eight transcriptome. About 797 and 1167 unigenes encoding ROS related proteins and transcription factors were identified. Of them, 11 and 16 ROS scavenging system related DEGs, and 29 and 15 transcription factors related DEGs were found in Baiyewuyueman and Kuishan’aijiaoheiye, respectively. Ten ROS-scavenging genes (*Cu/Zn*-*SOD*, *GST1*, *PODs*, *TrxR2*, *PrxR*, *FER3* and *NDPK*) showed higher expression levels in Cd-exposed seedings of Baiyewuyueman than those of Kuishan’aijiaoheiye. Four genes (*GPX*, *APX*, *GRX* and GST3) specifically expressed in Cd-free roots of Kuishan’aijiaoheiye. For transcription factors, *ERF12/13/22* and WRKY31 was up-regulated by Cd in Baiyewuyueman, while in Kuishan’aijiaoheiye, Cd induced down-regulations of bZIP, NAC and ZFP families. The results indicate that the two cultivars differed in the mechanism of ROS scavenging in response to Cd stress. Fe SOD1, POD A2/44/54/62 and GST1 may be responsible for the difference of Cd tolerance between Baiyewuyueman and Kuishan’aijiaoheiye.

## Introduction

Reactive oxygen species (ROS) are generated commonly as by-products from various metabolic processes of plants (i.e., photosynthesis and respiration)^1, 2^. The main ROS include singlet oxygen (^1^O_2_), superoxide anion (O_2_^−^), hydrogen peroxide (H_2_O_2_) and hydroxyl radical (HO·)^3^. ROS serve not only as dangerous molecules that damage proteins, lipids and DNA, but also as signaling/alarm molecules in regulation of biological processes such as biotic and abiotic stress responses, growth and development^2, 4^. Generally, the level of ROS in the different cellular compartments are maintained as a steady-state by antioxidant defense system including enzymatic and non-enzymatic mechanisms^1, 4^. The antioxidant enzymes include superoxide dismutase (SOD), catalase (CAT), glutathione peroxidase (GPX), ascorbate peroxidase (APX), peroxidase (POD) and glutathione reductase (GR), and the non-enzymatic antioxidants include glutathione (GSH), ascorbate (AsA) and others^5^. However, heavy metals can enhance generation of ROS in plants due to disruption of cellular homeostasis, which cause an imbalance between generation and scavenging of ROS, resulting in oxidative stress^4^. Therefore, it is of great importance to clarify the antioxidant defense mechanisms of plants in response to heavy metal stress.

Cadmium (Cd) is one of highly toxic heavy metals that can easily be absorbed by plants from soil and water. Excess Cd cause health hazard to all organisms through the food chain^6^. Cd can increase production of ROS by disturbing the antioxidative systems and damaging the electron transport systems, resulting in oxidative stress in plants^2, 7^. Thus, the genotypic difference of a plant species in cadmium tolerance was associated with the levels of oxidative stress and antioxidants^8, 9^. Wu *et al*.^9^ found that the high-Cd line (L351) of oilseed rape showed higher levels of expression in *BnFe*-*SOD*, *BnCAT*, *BnAPX*, *BcGR* and *BoDHAR* in the roots compared with low-Cd line (L338). Overexpression of the *SaCu/Zn SOD* in transgenic *Arabidopsis* plants resulted in an increase of Cd tolerance^10^. GSH accumulation occurred through enhanced gene expression of *LcGSHS* and the overexpression of *LcGSHS* exhibited higher tolerance to Cd stress in transgenic Arabidopsis than wild-types^11^. These results indicate that antioxidant systems play important roles in Cd tolerance.

Besides, many transcription factors (TFs) might function as ROS upstream regulators^12^, and have been extensively identified to improve plant tolerance to Cd stress. Overexpression of *ThbZIP1* in tobacco increased the activity of both POD and SOD under salt stress^13^. Overexpression of *TaWRKY*44 decreased H_2_O_2_ content, increased SOD, CAT, and POD activities, and upregulated the expression of some ROS-related genes in transgenic tobacco lines under osmotic stress condition^14^. Moreover, Hong *et al*.^15^ reported that *ZmWRKY4* might play a critical role in either regulating the *ZmSOD4* and *ZmcAPX* expression or cooperating with them in response to Cd stress and phytohormone. In spite of these findings, the integrated and comprehensive dissection for the molecular mechanism of Cd tolerance, especially in pak choi, is still undefined.

RNA-Seq is a very powerful technology for transcriptomics studies^16^. In recent years, RNA-seq has provided a useful tool for identification of related genes and their expression patterns in plant species responding to Cd stress^17–20^. Pak choi (*Brassica rapa* L. ssp. chinensis) is an important leafy vegetable crop. Variability among pak choi cultivars in Cd accumulation has been reported^21, 22^. Although several studies revealed that antioxidant enzymes play important roles in genotypic variation of Cd tolerance, understanding the antioxidative defense mechanisms in different pak choi cultivars is still unknown, especially at the molecular level.

Here, a comparative transcriptome analysis was performed in two pak choi cultivars, Baiyewuyueman (high Cd accumulator) and Kuishan’aijiaoheiye (low Cd accumulator) under Cd exposure using RNA-seq. The aims were: (i) to identify ROS scavenging-related differentially expressed genes (DEGs) between two cultivars; (ii) to identify DEGs encoding TFs involved in ROS scavenging of pak choi under Cd exposure; (iii) to investigate the molecular regulatory network of antioxidant defense system underlying the difference of Cd tolerance between two pak choi cultivars.

## Results

### Overview of transcriptome sequencing in pak choi

To establish an overall reference sequence database, eight cDNA libraries constructed from the roots of two pak choi cultivars(Cd/control RNA samples) were sequenced by RNA-seq. An approximate of 0.234 billion (B) and 0.224 billion (K) raw reads were generated, and near 0.227 billion and 0.217 billion clean reads were obtained from Baiyewuyueman and Kuishan’aijiaoheiye, respectively, with a total of 0.458 billion raw reads and 0.443 billion clean reads in this sequencing (Table 1). Then, totally 244,190 unigenes were assembled using the Trinity program. These mRNA transcriptome sequences formed the reference genome for identification of DEGs involved in antioxidant system of pak choi.

**Table 1:** Summary of Illumina transcriptome sequencing from pak choi roots.

Libraries	Raw Reads	Clean Reads	Clean reads in raw reads (%)	Unigene
B1_Cd_0_	66,471,522	64,624,428	97.22	
B2_Cd_0_	53,091,622	51,435,618	96.88	
B1_Cd_10_	64,130,424	61,972,662	96.64	
B2_Cd_10_	50,319,622	48,490,708	96.37	
subtotal	234,013,190	226,523,416	97.00	
K1_Cd_0_	55,192,566	53,300,252	96.57	
K2_Cd_0_	50,640,386	49,035,860	96.83	
K1_Cd_10_	55,041,300	52,835,866	95.99	
K2_Cd_10_	63,262,852	61,379,256	97.02	
subtotal	224,137,104	216,551,234	96.62	
total	458,150,294	443,074,650	96.71	244,190

### Functional annotation of the assembled unigenes in pak choi

**Figure 1:**
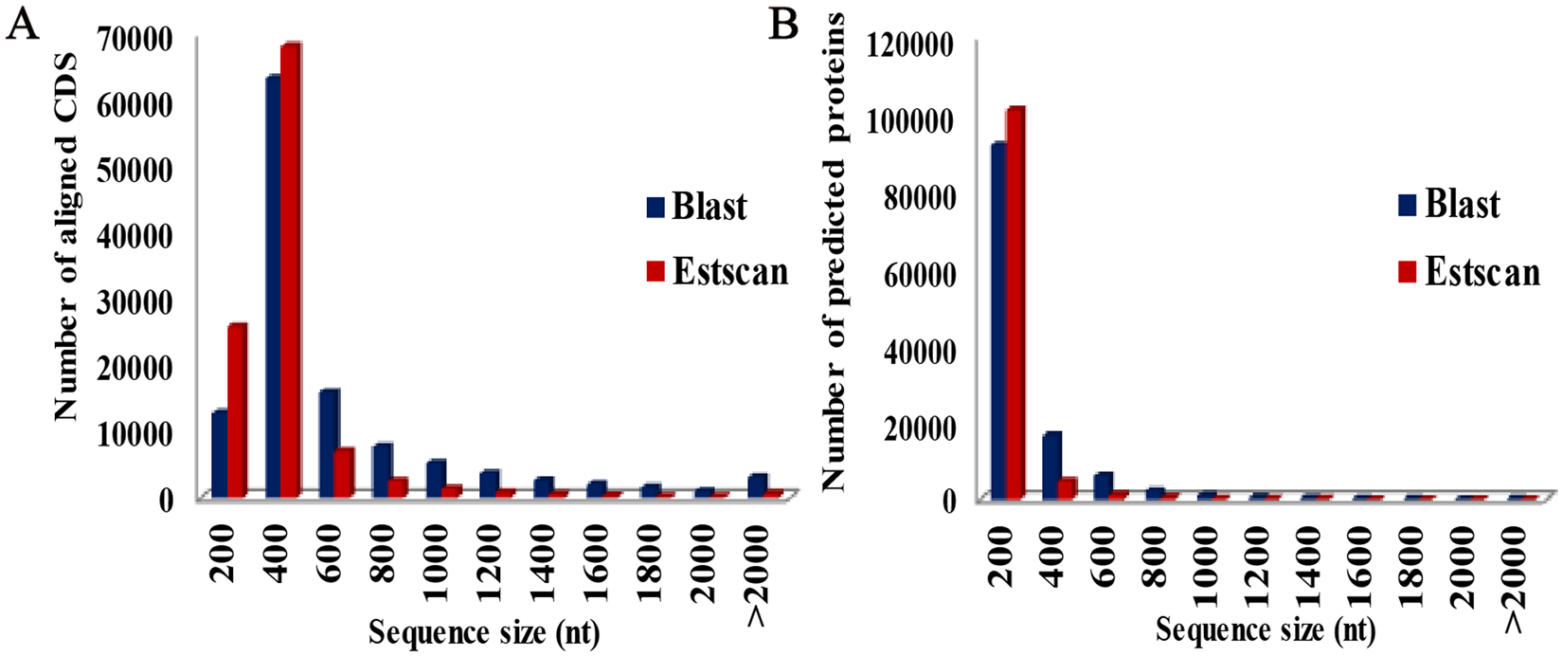
The length distribution of the coding sequence (CDS) and predicted proteins by BLASTx and Estcan software from the unigenes. Aligned CDS (**A**) and predicted proteins (**B**) by BLASTx and Estcan.

To further obtain information of unigene sequences from transcriptome, the unigene sequences were performed against public databases (Nr, Nt, Pfam, Swiss-Pot, KEGG, GO and KOG) using BLAST algorithm (*E*-value ≤ 10^−5^). The results of unigenes functional annotation were showed in Table S1. Based on Nr and Swiss-Pot databases, a total of 118,773 and 107,656 CDS were obtained and translated into peptide sequences by Blast and Estscan alignment, respectively. The length distribution of CDS and predicted proteins by BLAST and Estscan software were showed in Fig. 1.

**Figure 2:**
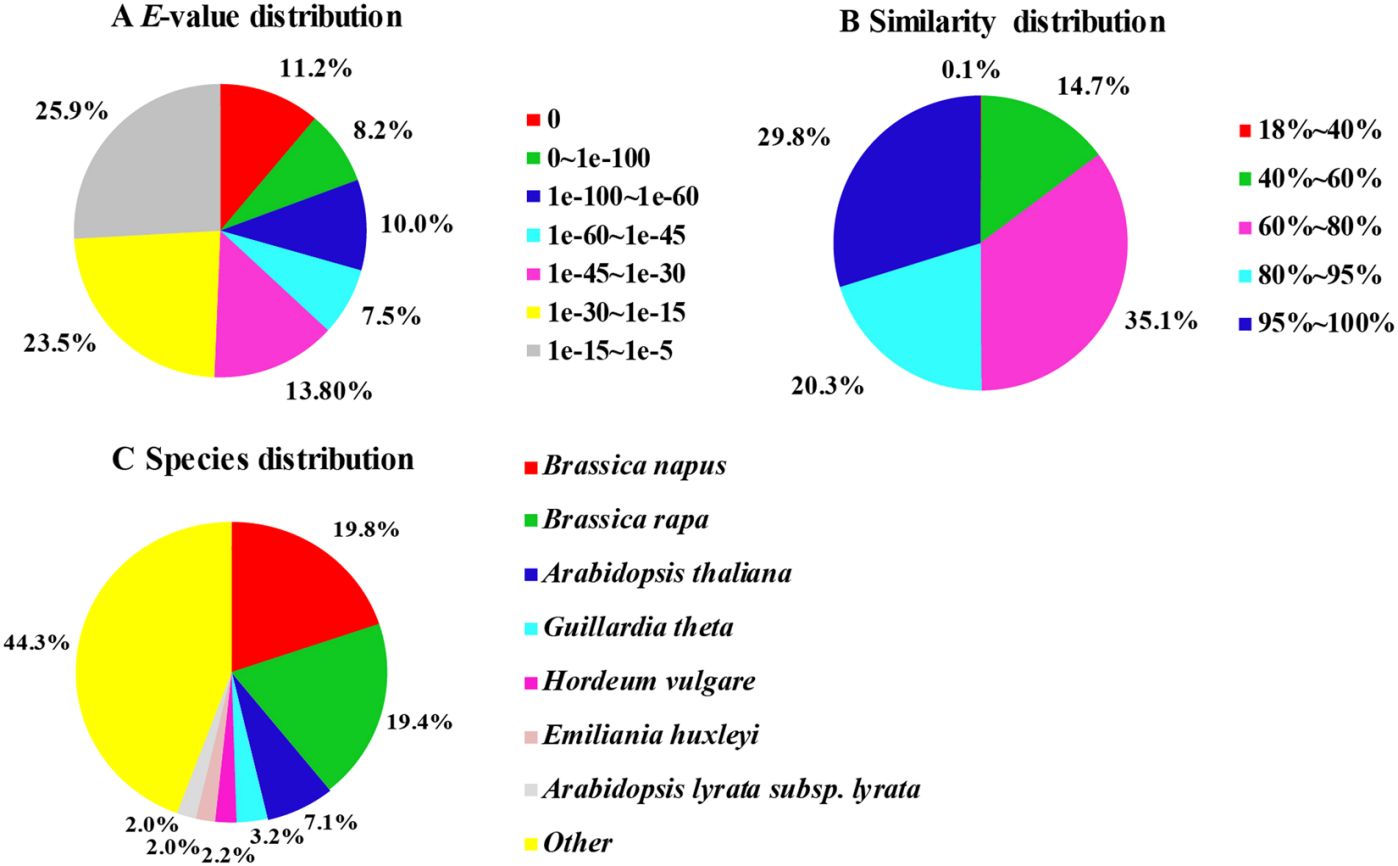
Characteristics of sequence homology of pak choi root BLAST against NCBI non-redundant (NR) database. (**A**) *E*-value distribution of BLAST hits for matched unigene sequences, using an *E*-value cutoff of 10^−5^. (**B**) Similarity distribution of top BLAST hits for each unigene. (**C**) Species distribution of the top BLAST hits.

In addition, we further analyzed the *E*-value, similarity and species distribution of the top hits in the Nr database, and the results were listed in Fig. 2. The *E*-value distribution of the top hits in the Nr database indicated that 50.7% of the mapped sequences have significant homology (*E*-value < 1.0e^−30^), whereas the other 49.4% of the moderate homology sequences varied from 1.0e^−5^ to 1.0e^−30^ (Fig. 2A). The similarity distribution displayed 50.1% of the query sequences have a similarity greater than 80%, while 49.9% of the hits have a similarity ranging from 18% to 80% (Fig. 2B). In species distribution, we found that the most annotated sequences were similar to *Brassica napus* (19.8%) and *Brassica rapa* (19.4%), followed by *Arabidopsis thaliana* (7.1%) *Guillardia theta* (3.2%), *Hordeum vulgare* (2.2%), *Arabidopsis lyrata* subsp. *Lyrata* (2.0%), and *Emiliania huxleyi* (2.0%) (Fig. 2C). The species distribution indicated a bias towards *Brassica napus* and *Brassica rapa*, suggesting that the unigene sequences of the pak choi were assembled and annotated properly in this study.

### Identification of ROS-mediated related genes in response to Cd exposure

**Figure 3:**
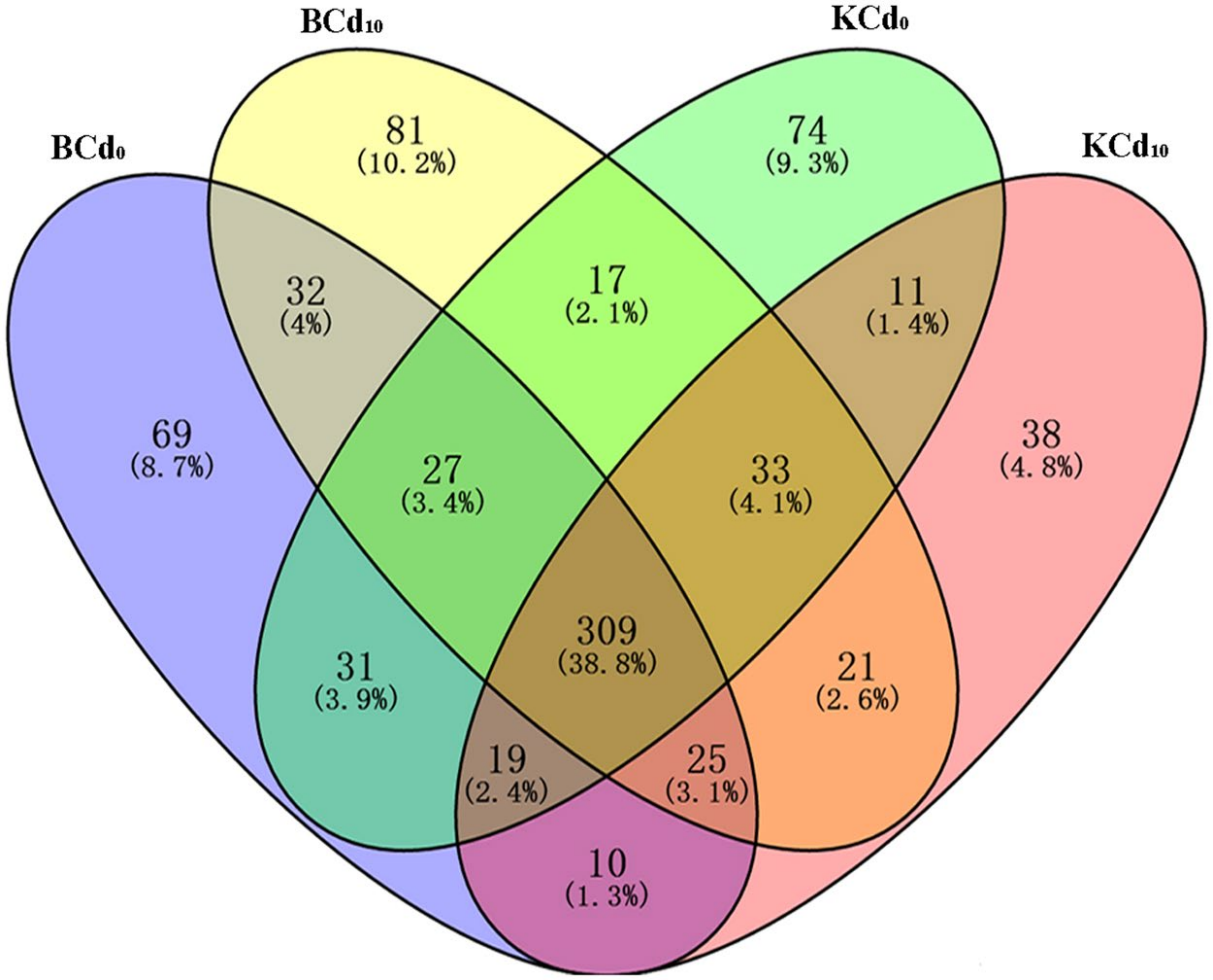
The all detected ROS-mediated related genes expression showed in Venn diagram.

Based on deep sequencing of the eight cDNA libraries GO and KEGG analysis, a total of 797 ROS-mediated related unigenes were identified in two pak choi cultivars (Table S2). Of them, 674 and 615 unigenes were detected in Baiyewuyueman and Kuishan’aijiaoheiye, respectively. The Venn diagram showed that there are 309 (309/797, 38.8%) unigenes that were shared by all four groups and 262 unigenes (69, 81, 74 and 38 unigenes in the BCd_0_, BCd_10_, KCd_0_ and KCd_10_, respectively) were specifically expressed in a single library. Additionally, totally 386 (386/657, 58.8%) unigenes synchronously expressed in BCd_0_ and KCd_0_, and 388 (388/623, 62.3%) unigenes in BCd_10_ and KCd_10_ (Fig. 3). Based on the unigenes FPKM values, the expressions of nine ROS related gene in Baiyewuyueman and Kuishan’aijiaoheiye were summarized in Table S3. Of them, 300 unigenes were showed similar expression patterns in Kuishan’aijiaoheiye and Baiyewuyueman under Cd treatment compared to their respective controls, while 190 unigenes showed opposing expression patterns.

### Identification of DEGs involved in ROS scavenging mechanism in response to Cd exposure

Among the 797 ROS-mediated related unigenes, a total of 11 (11 transcripts) and 15 DEGs (16 transcripts) were identified to show high similarity with ROS scavenging system-related genes in BCd_10_ vs. BCd_0_ and KCd_10_ vs. KCd_0_, respectively (Table S4A; Table 2). In Baiyewuyueman, most of DEGs (81.8%) including ferric reduction oxidase 2 (*FRO2*), glutathione S-transferase isoform 1 (*GST1*), protein disulfide isomerase 3 (*PDI3*), *Cu/Zn*-*SOD* (c111722_g2), *PODs* and peroxiredoxin (*PrxR*), were up-regulated by Cd, while only two genes such as thioredoxin-like 1-1 (*Trx1*-*1*) and NADPH-dependent thioredoxin reductase (*TrxR*) were down-regulated. In Kuishan’aijiaoheiye, most of DEGs (87.5%), such as *FRO2*, *Cu/Zn*-*SOD* (c111722_g1), ferritin-3 (*FER3*), glutathione gamma-glutamylcysteinyltransferase (*GGGT*), glutathione S-transferase (*GSTs*), glutaredoxin (*GRX*), nucleoside-diphosphate kinase (*NDPK*), thioredoxin domain-containing protein PLP3B (*PLP3B*), *GPX*, *APX*, and *TrxR2*, were down-regulated by Cd, and only two genes including Fe superoxide dismutase 1 (*Fe*-*SO797D1*) and *GST1* were up-regulated.

**Table 2:** The identified candidate genes involved in ROS scavenging system in response to Cd exposure in pak choi.

Unigene ID	Gene Description	Gene name	log_2_ Readcount ratio			*p*-adjusted		
			BCd10/BCd0	KCd10/KCd0	KCd10/BCd10	BCd_10_ vs. BCd_0_	KCd_10_ vs. KCd_0_	KCd_10_ vs. BCd_10_
c111359_g4	ADP/ATP translocase 1	*AAT1*	5.865347	−14.946	−5.82635	1	4.7274E-39	1
c111777_g1	Fe superoxide dismutase 1, Fe-Mn family	*Fe*-*SOD1*	1.194788	1.42877	1.163955	0.083021	3.4508E-06	5.0409E-08
c111722_g2	superoxide dismutase, Cu-Zn family	*Cu/Zn*-*SOD*	4.104719	2.9075	−1.20676	0.01282	0.94658	1
c111722_g1	superoxide dismutase, Cu-Zn family	*Cu/Zn*-*SOD*	0.002702	−1.6693	−1.04001	1	0.0052312	0.13019
c105537_g2	ferric reduction oxidase 2	*FRO2*	1.685025	−1.5766	−4.14407	8.9402E-08	0.024364	3.93E-47
c63214_g2	ferritin-3	*FER3*	5.865347	−13.365	−5.82635	1	8.2288E-12	1
c63214_g1	ferritin-3	*FER3*	0	−12.659	0	NA	4.1182E-08	NA
c82210_g1	glutathione gamma-glutamylcysteinyltransferase	*GGGT*	0	−13.565	0	NA	0.0073943	NA
c95296_g2	glutathione peroxidase	*GPX*	0	−11.086	0	NA	0.0054784	NA
c97139_g1	glutathione S-transferase	*GST*	0	−13.771	0	NA	0.0097912	NA
c71792_g1	glutathione S-transferase 3	*GST3*	0	−13.781	0	NA	0.040538	NA
c98684_g1	glutathione S-transferase isoform 1	*GST1*	3.084974	11.1703	−0.64776	0.015764	0.005515	1
c42503_g1	L-ascorbate peroxidase	*APX*	0	−13.942	0	NA	1.4913E-05	NA
c103054_g1	monothiol glutaredoxin	*GRX*	0	−12.597	0	NA	0.017912	NA
c72868_g1	nucleoside-diphosphate kinase	*NDPK*	5.535616	−14.701	−5.49449	1	0.019301	1
c105890_g1	peroxidase 44-like	*POD 44*	1.118188	0.32902	−1.34271	0.023056	1	0.00079376
c97892_g1	peroxidase 54	*POD 54*	1.026703	0.46609	−0.72778	0.020677	1	0.041487
c78850_g1	peroxidase 62	*POD 62*	1.588517	1.36876	−1.24846	7.8659E-07	1	0.022465
c97892_g2	peroxidase A2	*POD A2*	1.342933	0.65313	−1.48458	0.04055	1	0.00010904
c235353_g1	peroxiredoxin	*PrxR*	11.37772	0.0656	−0.35207	0.00034539	1	1
c59041_g1	thioredoxin domain-containing protein PLP3B	*PLP3B*	0	−13.808	0	NA	0.027499	NA
c166028_g1	protein disulfide isomerase family A, member 3	*PDI*	10.9317	−0.1886	−0.16004	0.010017	1	1
c104421_g2	thioredoxin-like 1-1	*Trx1*-*1*	−1.11759	−0.3378	0.550077	0.0022104	1	0.27375
c102953_g3	thioredoxin reductase 2	*TrxR2*	−0.88796	−12.814	−7.07945	1	0.014115	1
c104854_g1	NADPH-dependent thioredoxin reductase	*TrxR*	−16.3511	0	0	3.8393E-79	NA	NA

The *FRO2* (Fold change: −1.58), *FER3* (c63214_g2; Fold change: 13.34) and *NDPK* (Fold change: −14.70) were dramatically down-regulated by Cd exposure in Kuishan’aijiaoheiye, while in Baiyewuyueman, they were up-regulated. *Cu/Zn*-*SOD* (c111722_g1; Fold change: −1.67) was significantly down-regulated in Kuishan’aijiaoheiye, while in Baiyewuyueman, it was unchanged. *PrxR* was significantly up-regulated (Fold change: 11.38) in Baiyewuyueman, while in Kuishan’aijiaoheiye, it was unaffected. Moreover, all transcripts encoding *APX* (Fold change: −13.94), *GPX* (Fold change: −11.09), *GRX* (Fold change: −12.60), *PLP3B* (Fold change: −13.81) and *GGGT* (Fold change: −13.56) were specifically down-regulated by Cd in Kuishan’aijiaoheiye (Table 2).

In KCd_10_ vs. BCd_10_, a total of six DEGs were homologous with ROS scavenging system-related genes, including *PODs* (*44*, 54, 62 and *A2*), *FRO2* and *Fe*-*SOD1* (Table 2). Among them, all transcripts belonging to *PODs* and *FRO2* were down-regulated in KCd_10_ as compare to BCd_10_, while, only *Fe*-*SOD1* was up-regulated in KCd_10_.

### Cd stress regulated transcription factors

The regulation of Cd stress related genes expression can be achieved by transcription factors (TF) from different families such as myeloblastosis protein (MYB), basic leucine Zipper (bZIP), ethylene-responsive factor (ERF), heat shock transcription factor (HSF) and WRKY families^12^. In this study, a total of 1167 unigenes encoding proteins with homology to TFs belonging to 18 various families was identified from two cultivars (Table S5). Of them, 44 DEGs (BCd_10_ vs. BCd_0_, 29 unigenes; KCd_10_ vs. KCd_0_, 15 unigenes) were identified to have high similarity with TFs belonging to 11 various families under Cd exposure (Table S6). In Baiyewuyueman, three up-regulated transcripts and seven down-regulated transcripts were characterized as ERF TFs under Cd treatment. Moreover, one transcript of GATA (c112444_g1), and two transcripts of WRKY (c106672_g3 and c106672_g5) were up-regulated by Cd treatment, the remaining unigenes belonging to MYB, bZIP, basic helix–loop–helix (bHLH), nuclear factor Y (NF-Y), homeodomain-leucine zipper protein (HD-ZIP), no apical meristem (NAC) and zinc finger protein (ZFP) families were all down-regulated under Cd exposure. In Kuishan’aijiaoheiye, Cd-induced all unigenes belonging to bZIP, GATA, HD-ZIP, HSF, NAC and ZFP families were down-regulated. Furthermore, under Cd treatment, most of unigenes encoding TFs belonging to ERF, HD-ZIP, MYB and ZFP families were significant up-regulated, while, all unigenes belonging to NAC and WRKY dramatically trended downward in Kuishan’aijiaoheiye compared to Baiyewuyueman.

### Validation of ROS-scavenging genes by RT-qPCR

**Figure 4:**
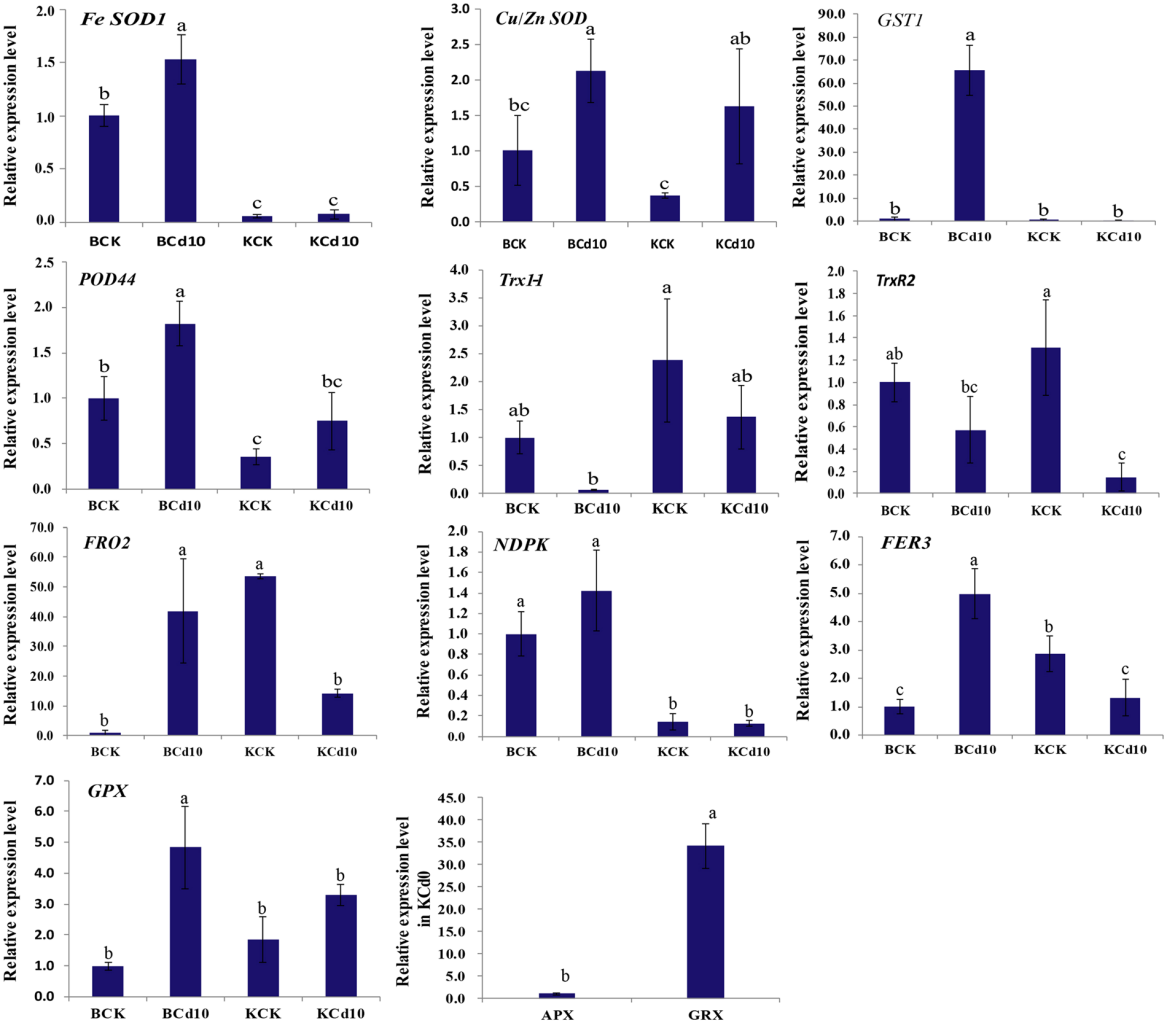
RT-qPCR analyses of 12 ROS scavenging-related genes under the control and Cd treatment in roots of two pak choi cultivars. Each bar represents the mean ± STD of triplicate assays. Values with different letters indicate significant differences at *P* < 0.05 according to Duncan’s multiple range tests.

To evaluate the reliability and validity of ROS-related antioxidant DEGs, a total of 12 DEGs were chosen and validated by RT-qPCR analysis. As shown in Fig. 4, all DEGs were differentially expressed between Baiyewuyueman and Kuishan’aijiaoheiye under Cd treatment. For both cultivars, the expression patterns of *Cu SOD* and *POD44* were significantly up-regulated by Cd exposure, and *Trx1*-*1* and *TrxR2* were down-regulated. *Fe SOD1*, *NDPK*, *GST1* and *GPX* were strongly up-regulated by Cd stress in Baiyewuyueman, while in Kuishan’aijiaoheiye, they were unaffected. *FRO2* and *FER3* were up-regulated by Cd in Baiyewuyueman, whereas they were down-regulated in Kuishan’aijiaoheiye. In the absence of Cd, *APX* and *GRX* were specifically expressed in Kuishan’aijiaoheiye. In the presence of Cd, the relative expression levels of 12 genes except *Trx1*-*1*, *APX* and *GRX*, were significantly higher in Baiyewuyueman than in Kuishan’aijiaoheiye. RT-qPCR results were generally similar to those of the RNA-Seq-based gene expression patterns (Table 2). However, *GPX* and *Fe SOD1* (KCd_10_ vs. BCd_10_) did not show consistent expression levels between RT-qPCR and Illumina sequencing data (Fig. 4). The discrepancies may be result from different sensitivity of the two techniques.

## Discussion

As reported, the genotypic difference in cadmium tolerance of plants was associated with the levels of oxidative stress and ROS scavenging antioxidants^7, 9^. Pak choi is one of the most marketable leaf vegetable crops, which showed a strong ability to accumulate cadmium^21, 22^. A previous study has indicated that Baiyewuyueman could accumulate more Cd in shoots than Kuishan’aijiaoheiye^22^, however, little is known about the molecular mechanisms of Cd tolerance in the two cultivars. In this study, a total of 797 unigenes encoding ROS-mediated related genes were identified in four groups (Fig. 3). Among these unigenes, 81 unigenes were specifically regulated by Cd exposure in Baiyewuyueman, while only 38 unigenes in Kuishan’aijiaoheiye (Fig. 3), implying that they might specifically regulated Cd-induced ROS accumulation and related oxidative damage at the corresponding cultivar. Furthermore, we found that 300 overlapping unigenes showed similar expression patterns in Kuishan’aijiaoheiye and Baiyewuyueman under Cd exposure (Table S3). These unigenes are not the key genes for regulating the difference of Cd tolerance in the two cultivars. Besides, 190 unigenes showed opposing expression patterns in Cd treatment (Table S3), suggesting that these unigenes might determine the changing of cadmium tolerance between two cultivars.

### ROS scavenging regulatory networks in response to Cd exposure in pak choi

Many studies showed that Cd can increase the production of ROS e.g., O_2_·^−^, H_2_O_2_ and OH·^5, 7, 23^, which could be eliminated by various ROS scavenging mechanisms including water-water cycle, AsA-GSH cycle, POD, GPX and PrxR/Trx pathways^24–27^.

Water-water cycle (O_2_·^−^-H_2_O_2_- H_2_O), mainly functioned by SOD and APX^27^. SOD (i.e., Cu/Zn SOD and Fe SOD) can convert O_2_·^−^into H_2_O_2_ and O_2_^9, 24^. APXs are directly involved in the converting H_2_O_2_ to H_2_O^26^. Overexpression of Fe/*Cu/Zn*-*SOD* in transgenic plants showed increased multiple stress tolerance^28, 29^. Overexpression of *SbpAPX* and *CaAPX* in transgenic tobacco enhanced abiotic stress tolerance^30, 31^. In this study, Cu/Zn SOD (c111722_g2) was up-regulated by Cd in Baiyewuyueman, while in Kuishan’aijiaoheiye, it was unaffected. Conversely, another unigene encoding Cu/Zn SOD (c111722_g1) was down-regulated by Cd in Kuishan’aijiaoheiye, while in Baiyewuyueman, it was not affected. In KCd_10_ vs. BCd_10_, Fe SOD1 showed higher expression levels in Cd-exposed seedings of Kuishan’aijiaoheiye than those of Baiyewuyueman (Table 2). The findings indicate that SOD may be involved in the difference of Cd tolerance between Kuishan’aijiaoheiye and Baiyewuyueman. Despite of this, we found that APX was down-regulated in Kuishan’aijiaoheiye, but not detected in Baiyewuyueman. The result suggests that the cultivar difference in Cd tolerance might be independent on water-water cycle.

AsA-GSH cycle, which is composed of monodehydroascorbate reductase (MDAR), GR and APX^27^. Although APX was down-regulated in Kuishan’aijiaoheiye, the expression levels of MDAR and GR were no significant difference in two cultivars under Cd treatment compared to their respective controls. Thus, the AsA-GSH cycle may not the key pathway for Cd detoxification in the two pak choi cultivars.

POD pathway can directly convert H_2_O_2_ into H_2_O and O_2_ in peroxisomes^26, 32^, and thus perform a significant function in responses to abiotic and biotic stresses. Overexpression of *MsPOD* in transgenic Arabidopsis exhibited resistance to H_2_O_2_ and NaCl-induced oxidative stress^33^. Increase of POD activity induced by Cd was reported in *Colocassia esculentum* root^34^. Here, four unigenes encoding POD A2/44/54/62 were strongly up-regulated by Cd in Baiyewuyueman, while in Kuishan’aijiaoheiye, they were unaffected (Table 2). POD A2/44/54/62 showed higher expression levels in Cd-exposed seedings of Baiyewuyueman than those of Kuishan’aijiaoheiye (Table 2). These results imply that POD pathway may be involved in the difference of Cd tolerance between Baiyewuyueman and Kuishan’aijiaoheiye. Specifically, Cd induced up-regulation of POD A2/44/54/62 may enhance H_2_O_2_ scavenging and thereby, contributing to Cd tolerance in Baiyewuyueman.

GPX pathway (including GPX, GRX and GST) is another process that convert H_2_O_2_ to H_2_O in plants depending on GSH^26, 35, 36^. There is evidence that transgenic plants overexpressing GST and GPX genes enhanced the stress tolerance^37, 38^. GRX is also involved in the protection of protein, enhancing the tolerance of plants under different abiotic stress^39, 40^. In the current study, GPX, GSTs (c71792_g1, c97139_g1) and GRX were down-regulated by Cd in Kuishan’aijiaoheiye, while in Baiyewuyueman, they were not detected regardless of Cd treatments (Table 2). The results indicate that the GPX pathway may not be involved in the mechanism of ROS scavenging for Baiyewuyueman, while the sensitivity to Cd in Kuishan’aijiaoheiye may be resulted from the down-regulation of GPX, GRX and GST under Cd stress. Additionally, Cd induced up-regulation of GST1 (c98684_g1) in both cultivars, and the expression were higher in Baiyewuyueman than in Kuishan’aijiaoheiye (Table 2 and Fig. 4), suggesting that GST1 may be involved in the difference of Cd tolerance between Baiyewuyueman and Kuishan’aijiaoheiye.

PrxR/Trx pathway is a NADPH oxidoreductase pathway, which has been confirmed involved in the regulation of the H_2_O_2_ to H_2_O via PrxR in conjunction with TrxR and Trx^32, 41^. The unigene encoding PrxR was up-regualted by Cd in Baiyewuyueman, while in Kuishan’aijiaoheiye, it was unchanged. Overexpressing AlrT4642, a novel PrxR-like protein, made *Anabaena* cells more resistant to H_2_O_2_^42^. Trx have been confirmed to protect protein and enhance plant tolerance under different abiotic stress^39, 40^. TrxR (c104854_g1) and Trx1-1 were down-regulated by Cd in Baiyewuyueman, while in Kuishan’aijiaoheiye, they were unchanged. TrxR2 (c102953_g3) was down-regualted by Cd in Kuishan’aijiaoheiye, while in Baiyewuyueman, it was unchanged (Table 2). The current results suggest that Cd induced up-regulation of PrxR may contribute to Cd tolerance in Baiyewuyueman.

In addition, other ROS detoxification genes such as ferritins (FERs) and NDPK were also identified in pak choi. The unigenes encoding FER3 (c63214_g1 and c63214_g2) and NDPK (c72868_g1) were significantly down-regulated by Cd in Kuishan’aijiaoheiye, while in Baiyewuyueman, they were not affected (Table 2). The results suggest that *FER3* and *NDPK* may not be involved in the mechanism of ROS scavenging for Baiyewuyueman, while in Kuishan’aijiaoheiye, Cd-induced down-regulation of these genes may result in Cd sensitivity. Ferritins (FERs), as iron-storage proteins, has been reported to sequester Fe^2+^ and to prevent the formation of OH· via the Haber-Weiss or Fenton reactions^4, 43^. Overexpression of ferritin significantly improved abiotic stress tolerance in grapevine (*MsFER*)^44^ and wheat (*TaFER*-*5B*)^43^. NDPK is associated with tolerance to abiotic stresses by the regulation of H_2_O_2_-mediated mitogen-activated protein kinase (MAPK) signaling^45^. Transgenic plants overexpressing *PtNDPK2* and *AtNDPK3* exhibit increased tolerance to abiotic stresses^46, 47^.

### Transcription factors are involved in ROS scavenging under Cd stress

Transcription factors, such as bHLH, bZIP, ERF, ZFP, WRKY, NAC and MYB TFs could protect cells against oxidative damage by triggering the activation of the ROS related genes expression^15, 48–50^. Our results showed that five unigenes encoding ERF12/13/22 and WRKY31 (c106672_g3 and c106672_g5) were up-regulated by Cd in Baiyewuyueman, whereas these TFs were not differentially expressed in Kuishan’aijiaoheiye exposed to different Cd (Table S6). The results suggest that the up-regulation of WRKY31 and ERF12/13/22 TFs may be involved in the mechanism of ROS scavenging for Baiyewuyueman. WRKY TFs are mainly involved in the response to abiotic and biotic stresses including Cd^51^. *ZmWRKY4* overexpressing in maize can elevate the expression of SOD and APX under Cd stress^15^. ThWRKY7 is an upstream regulator of ThVHAc1, and overexpression of ThWRKY7 in transgenic *Tamarix hispida* enhanced the activities of SOD, POD, GST, and GPX^49^. Overexpression of *GmWRKY31* in transgenic soybean enhanced resistance to *Phytophthora sojae* by regulating *GmNPR1*, which is associated with resistance to *P*. *sojae* by regulating pathogenesis-related genes^51^. ERF TFs could regulate the transcription of ROS metabolic enzymes to improve plant ROS tolerance^48, 52^. Zhang *et al*.^48^ reported that overexpression of *TERF1*,elevated the transcription of *NtGPX*, which may play key role in the regulation of ROS scavenging under Cd stress.

Cd exposure significantly down-regulated some TFs in Kuishan’aijiaoheiye, including bZIPs (c86110_g1, c95948_g2 and c106035_g1), NAC69 and ZFPs (c113235_g4, c65518_g1, c42694_g1, c88098_g1, c106940_g2 and c92024_g2), while in Baiyewuyueman, they were unaffected (Table S6). Wang *et al*. found that overexpression of *ThbZIP1* in tobacco enhanced the activity of both POD and SOD, and increased the content of soluble sugars and soluble proteins under salt stress conditions^13^. In *Arabidopsis*, ZAT6 belonging to ZFP family were shown to positively regulate Cd tolerance by the regulation of the *GSH1* expression^53^. Overexpression of *SlNAM1*, belonging to the NAC TF family, improved the osmolytes contents and reduced the H_2_O_2_ and O_2_^−^ contents under low temperature, which contribute to improving chilling stress tolerance in transgenic tobacco^54^. NAC69 is associated with plant adaptation to abiotic and biotic stresses. Overexpression of *TaNAC69*-*1* enhanced the expression of some stress up-regulated genes such as glyoxalase I family protein (detoxification function) and hydroxyphenylpyruvate dioxygenase (antioxidant function) in transgenic wheat, suggesting that *TaNAC69* is involved in regulating the drought tolerance in bread wheat^55^. Therefore, it seems that the sensitivity to Cd in Kuishan’aijiaoheiye may be resulted from the down-regulation of *bZIP*, *NAC* and *ZFP* TFs under Cd stress, while for Baiyewuyueman, these TFs may not be involved in the mechanism of ROS scavenging.

## Conclusions

**Figure 5:**
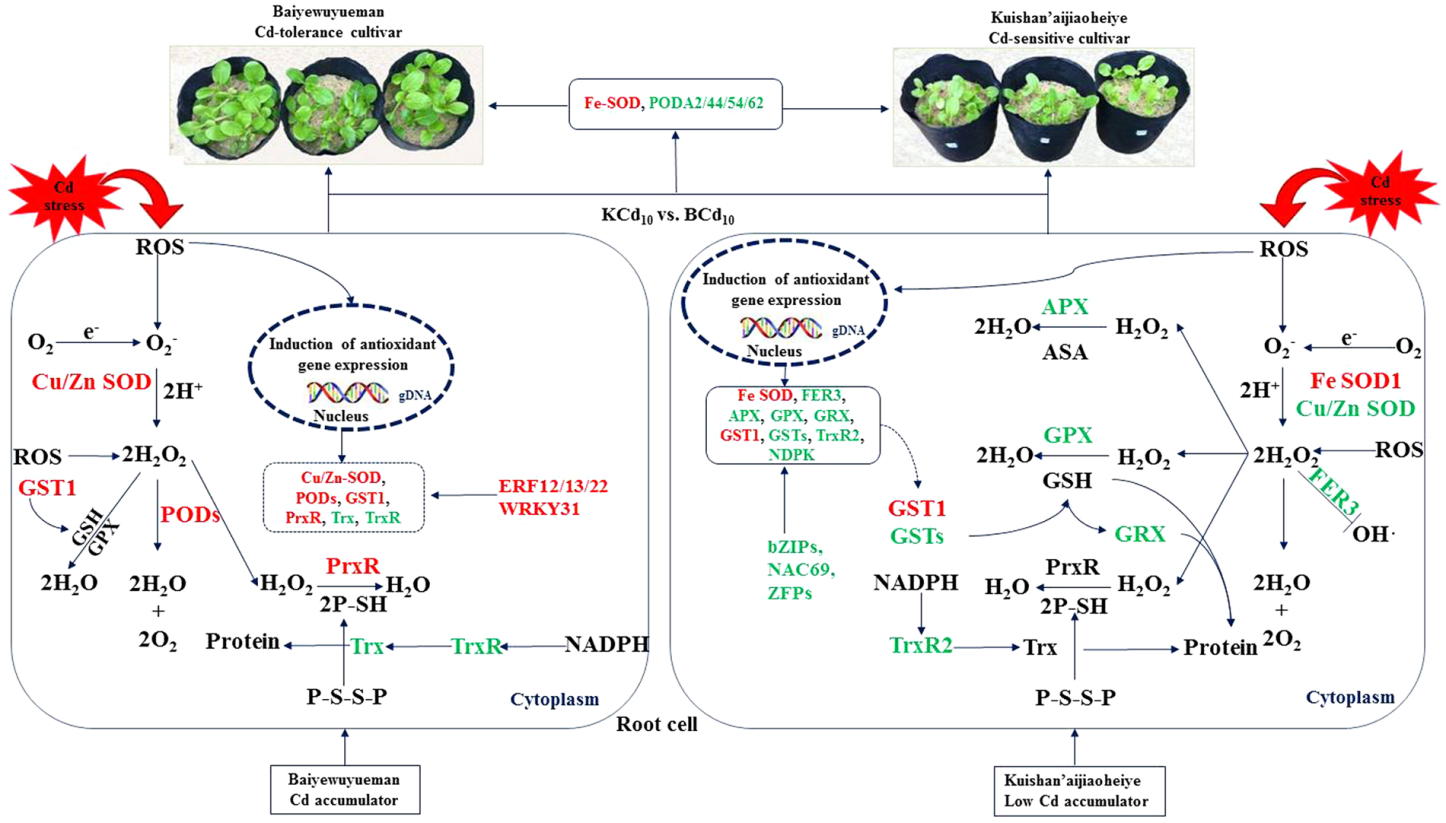
The putative model of regulatory networks associated with ROS scavenging system in response to Cd exposure in roots of two pak choi cultivars. Red fonts indicate the genes up-regulated, green fonts indicate the genes down-regulated in two cultivars exposed to Cd as compared to their respective controls.

In summary, the two cultivars differed in the mechanism of ROS scavenging in response to Cd stress (Fig. 5). For Baiyewuyueman, the POD and PrxR/Trx pathways were involved, which was regulated by TFs such as ERF12/13/22 and WRKY31. For Kuishan’aijiaoheiye, the water-water cycle and GPX pathways might be associated with its sensitivity to Cd, which was regulated by TFs including bZIP, NAC69 and ZFP. Among these DEGs, Fe SOD1, POD A2/44/54/62 and GST1 may be responsible for the difference of Cd tolerance between Baiyewuyueman and Kuishan’aijiaoheiye.

## Methods

### Plant materials and Cd treatment

Two pak choi cultivars, Baiyewuyueman (B, high-Cd cultivar) and Kuishan’aijiaoheiye (K, low-Cd cultivar), were used in this study. The seeds were sown in sand with spiked with 0 (Cd_0_) and 10 mg/kg Cd (Cd_10_) as CdCl_2_•2.5H_2_O as previously described^22^, and were cultivated in a growth chamber with 16 h light at 25 °C and 8 h darkness at 16 °C.

Root samples for RNA-seq and RT-qPCR analysis were collected separately at four weeks after seedling emergence. Multiple independent biological replicates, each containing a pool of six different plants, were sampled for RNA-Seq (two biological replicates) and RT-qPCR validation (three biological replicates). All samples were immediately frozen in liquid nitrogen and stored at −80 °C.

### RNA isolation, cDNA library construction and sequencing

Total RNA was extracted using Trizol^®^ Reagent (Invitrogen) from a total of eight individual samples. Approximately 1.5 µg of the extracted RNA per sample was used as input material for cDNA library construction and subsequent Illumina sequencing. The eight cDNA libraries (B1_Cd_0_, B2_Cd_0_, B1_Cd_10_, B2_Cd_10_, K1_Cd_0_, K2_Cd_0_, K1_Cd_10_ and K2_Cd_10_) were generated using NEBNext^®^ Ultra^TM^ RNA Library Prep Kit for Illumina^®^ (NEB, USA) following the manufacturer’s instructions^56^: (i) mRNA was purified using poly-T oligo-attached magnetic beads; (ii) fragmentation was carried out using divalent cations under elevated temperature in NEBNext; (iii) first strand cDNA was synthesized using random hexamer primer and M-MuLV Reverse Transcriptase (RNase H^−^); (iv) after second strand cDNA synthesis and adaptor ligation, the cDNA fragments were isolated and purified with AMPure XP system (Beckman Coulter, Beverly, USA); (v) Then PCR amplifications were selected as templates to create the final cDNA library, and library quality was assessed on the Agilent Bioanalyzer 2100 system. The resulting per cDNA library was sequenced using the Illumina Hiseq^TM^ 2500 platform following the manufacturer’s recommendations at Novogene Bioinformatics Institute (Beijing, China).

### Transcriptome sequencing results analysis

The sequences were assembled as previously described^56, 57^. The assembled unigenes were annotated by BLAST alignment to public protein and nucleotide databases including NCBI non-redundant protein (Nr) and non-redundant nucleotide sequences (Nt), Protein family (Pfam), euKaryotic Ortholog Groups (KOG), Swiss-Prot, Kyoto Encyclopedia of Genes and Genomes (KEGG) and Gene Ontology (GO) with an *E*-value cutoff of 10^−5^. The best-aligning results from Nr and Swiss-Prot databases were taken to decide the coding region sequences of unigenes. If the results from two databases conflicted with each other, a priority order of Nr and Swiss-Prot was considered. Meanwhile, if the unigene sequences could not be aligned to any one of database, the Estscan (3.0.3) software was used to predict the coding sequence (CDS) and their orientations. In addition, the GO functional annotation of unigenes were gained using Blast2GO program, and GO functional classification were obtained to classify the possible functions of the unigenes based on Nr annotations using WEGO software.

### Quantification of gene expression levels and identification of DEGs

The gene expression levels were estimated by RSEM^58^ for each sample: (i) the clean reads were mapped back onto the assembled transcriptome reference sequences; (ii) the readcount for each gene was obtained from the mapping results; (iii) and then the gene expression level of each gene was normalized to FPKM (Fragments Per Kilobase of transcript per Millions fragments) based on the number of readcount. Differential expression analysis of two groups was performed using the DESeq R package (1.10.1). The threshold for differential expression was set at adjusted *P*-value < 0.05 using the Benjamini and Hochberg’s approach.

### RT-qPCR analysis

Three biological replications with three technique replications of total RNA were used for RT-qPCR analysis. First strand cDNA fragments were synthesized using the PrimeScript^®^ RT reagent kit (Takara, Dalian, China). RT-qPCR was performed on an ABI 7300 (Applied Biosystems, Foster City, CA, USA) using a SYBR Premix EX Taq kit (Takara) in a 20 μl reaction mixtures. The PCR reaction protocol was 95 °C for 30 s, 40 cycles of 95 °C for 5 s and 60 °C for 30 s. The fluorescence was measured via a 65–95 °C melting curve. The specific primers for RT-qPCR were designed using Beacon Designer 7.0 software (Premier Biosoft International, USA) (Table S7). The relative expression level of the selected genes using the *Actin* gene as the internal control gene was calculated using ratio = 2^−ΔΔ*C*T^.

### Data deposition

The four data sets of RNA-sequencing are available at the NCBI Short Read Archive (SRA) with the GenBank accession No.: SRS1876732, SRS1876733, SRS1876734 and SRS1876738.

## Electronic supplementary material

Supplementary information

Table S1

Table S2

Table S3

Table S4

Table S5

Table S6

Table S7

## References

[1] Apel K, Hirt H (2004). Reactive oxygen species: metabolism, oxidative stress, and signal transduction. Annu Rev Plant Biol.

[2] Gupta DK (2016). NADPH oxidases differentially regulate ROS metabolism and nutrient uptake under cadmium toxicity. Plant Cell Environ.

[3] Martins LL (2011). Oxidative stress induced by cadmium in *Nicotiana tabacum* L.: effects on growth parameters, oxidative damage and antioxidant responses in different plant parts. Acta Physiol Plant.

[4] Sytar O (2013). Heavy metal-induced oxidative damage, defense reactions, and detoxification mechanisms in plants. Acta Physiol Plant.

[5] Gupta, D. K., Palma, J. M. & Corpas, F. J. *Reactive Oxygen Species and Oxidative Damage in Plants under Stress*. (ed. Gupta, D. K.) **74**, 621 (Springer-Verlag: Berlin, 2015).

[6] Shahid M, Dumat C, Khalid S, Niazi NK, Antunes PMC (2016). Cadmium bioavailability, uptake, toxicity and detoxification in soil-plant system. Rev Environ Contam Toxicol.

[7] Romero-Puertas MC (2004). Cadmium-induced subcellular accumulation of O_2_^·−^ and H_2_O_2_ in pea leaves. Plant Cell Environ.

[8] Tiryakioglu M, Eker S, Ozkutlu F, Husted S, Cakmak I (2006). Antioxidant defense system and cadmium uptake in barley genotypes differing in cadmium tolerance. J Trace Ele Med Bio.

[9] Wu Z (2015). Antioxidant enzyme systems and the ascorbate-glutathione cycle as contributing factors to cadmium accumulation and tolerance in two oilseed rape cultivars (*Brassica napus* L.) under moderate cadmium stress. Chemosphere.

[10] Li Z (2017). Overexpressing the Sedum alfredii Cu/Zn Superoxide Dismutase Increased Resistance to Oxidative Stress in TransgenicArabidopsis. Front Plant Sci.

[11] Guan C (2015). A GSHS-like gene from Lycium chinense maybe regulated by cadmium-induced endogenous salicylic acid and overexpression of this gene enhances tolerance to cadmium stress in Arabidopsis. Plant Cell Rep.

[12] Chan Z, Yokawa K, Kim WY, Song CP (2016). Editorial: ROS Regulation during Plant Abiotic Stress Responses. Front Plant Sci.

[13] Wang YC (2010). A novel bZIP gene from Tamarix hispida mediates physiological responses to salt stress in tobacco plants. J Plant Physiol.

[14] Xiatian W (2015). Expression of TaWRKY44, a wheat WRKY gene, in transgenic tobacco confers multiple abiotic stress tolerances. Front Plant Sci.

[15] Hong C (2016). The role of ZmWRKY4 in regulating maize antioxidant defense under cadmium stress. Biochem Biophys Res Commun.

[16] Liu MM, Xing YM, Zhang DW, Guo SX (2015). Transcriptome analysis of genes involved in defence response in *Polyporus umbellatus* with *Armillaria mellea* infection. Sci Rep.

[17] Shi H (2015). Comparative physiological, metabolomic, and transcriptomic analyses reveal mechanisms of improved abiotic stress resistance in bermudagrass [*Cynodon dactylon* (L). Pers.] by exogenous melatonin. J Exp Bot.

[18] Sun M, Li YT, Liu Y, Lee SC, Wang L (2016). Transcriptome assembly and expression profiling of molecular responses to cadmium toxicity in hepatopancreas of the freshwater crab *Sinopotamon henanense*. Sci Rep.

[19] Gao J (2015). Transcriptome sequencing and differential gene expression analysis in *Viola yedoensis* Makino (Fam. Violaceae) responsive to cadmium (Cd) pollution. Biochem Biophys Res Commun.

[20] Wang Z (2016). RNA-seq analysis revealed ROS-mediated related genes involved in cadmium detoxification in the razor clam *Sinonovacula constricta*. Fish Shellfish Immunol.

[21] Chen Y (2012). Cadmium accumulation in different pakchoi cultivars and screening for pollution-safe cultivars. J Zhejiang Univ Sci B.

[22] Xia S, Deng R, Zhang Z, Liu C, Shi G (2016). Variations in the accumulation and translocation of cadmium among pak choi cultivars as related to root morphology. Environ Sci Pollut Res.

[23] Anjum NA, Umar S, Iqbal M, Khan NA (2011). Cadmium causes oxidative stress in mung bean by affecting the antioxidant enzyme system and ascorbate-glutathione cycle metabolism. Russ J Plant Physiol.

[24] Gratão PL (2015). Cadmium stress antioxidant responses and root-to-shoot communication in grafted tomato plants. Biometals.

[25] Shahabivand S (2016). Antioxidant activity and gene expression associated with cadmium toxicity in wheat affected by mycorrhizal fungus. Zemdirbyste-agriculture.

[26] Karuppanapandian T, Juncheol M, Changsoo K, Manoharan K, Wook K (2011). Reactive oxygen species in plants: their generation, signal transduction, and scavenging mechanisms. Aust J Crop Sci.

[27] Fan, Y., Jayakumar, B., Zhou, M. & Sergey, S. ROS Production, Scavenging, and Signaling under Salinity Stress. *Managing Salt Tolerance in Plants*: *Molecular and Genomic Perspectives* (ed. Wani, S. H. & Hossain, M. A.) 187–199 (CRC press, 2015).

[28] Lee SH (2008). Simultaneous overexpression of both CuZn superoxide dismutase and ascorbate peroxidase in transgenic tall fescue plants confers increased tolerance to a wide range of abiotic stresses. J Plant Physiol.

[29] Gao X (2016). Transgenic *NfFeSOD Sedum alfredii* plants exhibited profound growth impairments and better relative tolerance to long-term abiotic stresses. Plant Biotechnol Rep.

[30] Singh N, Mishra A, Jha B (2014). Over-expression of the peroxisomal ascorbate peroxidase (*SbpAPX*) gene cloned from halophyte *Salicornia brachiata* confers salt and drought stress tolerance in transgenic tobacco. Mar Biotechnol.

[31] Wang, J. *et al*. Overexpression of CaAPX induces orchestrated reactive oxygen scavenging and enhances cold and heat tolerances in tobacco. *BioMed Res Int* **2017** (2017). 10.1155/2017/4049534 PMC5366785 28386551

[32] Mittler R, Vanderauwera S, Gollery M, Breusegem FV (2004). Reactive oxygen gene network of plants. Trends Plant Sci.

[33] Teng, K. *et al*. Expression of an alfalfa (*Medicago sativa* L.) peroxidase gene in transgenic *Arabidopsis thaliana* enhances resistance to NaCl and H_2_O_2_. *Genet Mol Res* **15** (2016). 10.4238/gmr.15028002 27323080

[34] Patel MJ, Patel JN, Subramanian RB (2005). Effect of cadmium on growth and the activity of H_2_O_2_ scavenging enzymes in *Colocassia esculentum*. Plant Soil.

[35] Wrzaczek M, Brosché M, Kangasjärvi J (2013). ROS signaling loops-production, perception, regulation. Curr Opin Plant Biol.

[36] Anjum NA (2013). *Eriophorum angustifolium* and *Lolium perenne* metabolic adaptations to metals- and metalloids-induced anomalies in the vicinity of a chemical industrial complex. Environ Sci Pollut Res.

[37] Roxas VP, Lodhi SA, Garrett DK, Mahan JR, Allen RD (2000). Stress tolerance in transgenic tobacco seedlings that overexpress glutathione S-transferase/glutathione peroxidase. Plant Cell Physiol.

[38] Yang G (2016). In planta characterization of a tau class glutathione S-transferase gene from Juglans regia (JrGSTTau1) involved in chilling tolerance. Plant Cell Rep.

[39] Rouhier N (2005). Identification of plant glutaredoxin targets. Antioxid Redox Signal.

[40] Meyer Y, Belin C, Delormehinoux V, Reichheld JP, Riondet C (2012). Thioredoxin and glutaredoxin systems in plants: molecular mechanisms, crosstalks, and functional significance. Antioxid Redox Signal.

[41] Choi JH (2002). Overexpression of mitochondrial thioredoxin reductase and peroxiredoxin III in hepatocellular carcinomas. Anticancer Res.

[42] Tailor V, Ballal A (2015). Over-expression of Alr4642, a novel Prx-like peroxiredoxin, defends the cyanobacterium *Anabaena* PCC7120 from oxidative stress. J Appl Phycol.

[43] Zang X (2017). Overexpression of wheat ferritin gene *TaFER*-*5B* enhances tolerance to heat stress and other abiotic stresses associated with the ROS scavenging. Bmc Plant Biol.

[44] Zok A (2010). Effect of *Medicago sativa* ferritin gene on stress tolerance in transgenic grapevine. Plant Cell Tissue Org Cult.

[45] Moon H (2003). NDP kinase 2 interacts with two oxidative stress-activated MAPKs to regulate cellular redox state and enhances multiple stress tolerance in transgenic plants. P Natl Acad Sci USA.

[46] Zhang J (2017). Functional analyses of NDPK2 in *Populus trichocarpa* and overexpression of *PtNDPK2* enhances growth and tolerance to abiotic stresses in transgenic poplar. Plant Physiol Bioch.

[47] Liu H (2015). Stress signaling in response to polycyclic aromatic hydrocarbon exposure in *Arabidopsis thaliana* involves a nucleoside diphosphate kinase, NDPK-3. Planta.

[48] Zhang H (2016). Ethylene response factor TERF1, regulated by ethylene-insensitive 3-like Factors, functions in reactive oxygen species (ROS) scavenging in tobacco (*Nicotiana tabacum* L.). Sci Rep.

[49] Yang G (2016). Overexpression of *ThVHAc1* and its potential upstream regulator,*ThWRKY7*, improved plant tolerance of cadmium stress. Sci Rep.

[50] Wu H (2012). Co-overexpression FIT with *AtbHLH38* or *AtbHLH39* in Arabidopsis-enhanced cadmium tolerance via increased cadmium sequestration in roots and improved iron homeostasis of shoots. Plant Physiol.

[51] Fan S (2017). GmWRKY31 and GmHDL56 enhances resistance to *Phytophthora sojae* by regulating defense-related gene expression in soybean. Front Plant Sci.

[52] Sewelam N (2013). Ethylene response factor 6 is a regulator of reactive oxygen species signaling in Arabidopsis. PloS One.

[53] Chen J (2016). Zinc-finger transcription factor ZAT6 positively regulates cadmium tolerance through the glutathione-dependent pathway in Arabidopsis. Plant Physiol.

[54] Li XD (2016). Overexpression of a novel NAC-type tomato transcription factor, *SlNAM1*, enhances the chilling stress tolerance of transgenic tobacco. J Plant Physiol.

[55] Xue GP (2011). Overexpression of *TaNAC69* leads to enhanced transcript levels of stress up-regulated genes and dehydration tolerance in bread wheat. Mol Plant.

[56] Hu L (2015). RNA-seq for gene identification and transcript profiling in relation to root growth of bermudagrass (*Cynodon dactylon*) under salinity stress. BMC genomics.

[57] Grabherr MG (2011). Full-length transcriptome assembly from RNA-Seq data without a reference genome. Nat Biotechnol.

[58] Li B, Dewey CN (2011). RSEM: accurate transcript quantification from RNA-Seq data with or without a reference genome. BMC bioinformatics.

