# Supplementary material for: Comparative transcriptomic analysis reveals the roles of ROS scavenging genes in response to cadmium in two pak choi cultivars
Source: Sci Rep. 2017 Aug 23;7:9217. doi: 10.1038/s41598-017-09838-2 (PMC5569009; doi:10.1038/s41598-017-09838-2)
Supplement: Supplementary file 1 — Supplementary information [file 41598_2017_9838_MOESM1_ESM.pdf]

Supplementary information

Comparative transcriptomic analysis reveals the roles of ROS scavenging antioxidant defense genes in response to cadmium in two pak choi cultivars

Rugang Yu[1], Yunshu Tang[1], Caifeng Liu[1], Xueling Du[1], Chunmei Miao[1], Gangrong Shi[1*]

Supplementary Tables

Supplementary Table S1. The functional annotation results of 244,190 unigenes in pak choi.

Supplementary Table S2. Identification and quantification of ROS-mediated related genes in Baiyewuyueman and Kuishan’aijiaoheiye.

Supplementary Table S3. A summary of nine ROS- mediated related gene expression patterns in Baiyewuyueman and Kuishan’aijiaoheiye.

Supplementary Table S4A. The DEGs involved in ROS scavenging mechanism in two pakchoi cultivars under Cd exposure.

Supplementary Table S4B. The analysis results of DEGs involved in ROS scavenging mechanism in two cultivars under Cd exposure.

Supplementary Table S5. A summary of the assembled unigenes encoding transcription factors in Baiyewuyueman and Kuishan’aijiaoheiye.

Supplementary Table S6. The DEGs encoding transcription factors in two pakchoi cultivars under Cd exposure.

Supplementary Table S7. The specific primer sequences of 12 ROS scavenging-related DEGs validated by RT-qPCR analysis.
